# Patterns of Natural Selection on Mitochondrial Protein-Coding Genes in Lungless Salamanders: Relaxed Purifying Selection and Presence of Positively Selected Codon Sites in the Family Plethodontidae

**DOI:** 10.1155/2021/6671300

**Published:** 2021-04-09

**Authors:** Ryosuke Kakehashi, Atsushi Kurabayashi

**Affiliations:** ^1^Faculty of Bio-Science, Nagahama Institute of Bio-Science and Technology, Shiga 526-0829, Japan; ^2^Unit for Environmental Sciences and Management, North-West University, Potchefstroom 2520, South Africa

## Abstract

There are two distinct lungless groups in caudate amphibians (salamanders and newts) (the family Plethodontidae and the genus *Onychodactylus*, from the family Hynobiidae). Lunglessness is considered to have evolved in response to environmental and/or ecological adaptation with respect to oxygen requirements. We performed selection analyses on lungless salamanders to elucidate the selective patterns of mitochondrial protein-coding genes associated with lunglessness. The branch model and RELAX analyses revealed the occurrence of relaxed selection (an increase of the dN/dS ratio = *ω* value) in most mitochondrial protein-coding genes of plethodontid salamander branches but not in those of *Onychodactylus*. Additional branch model and RELAX analyses indicated that direct-developing plethodontids showed the relaxed pattern for most mitochondrial genes, although metamorphosing plethodontids had fewer relaxed genes. Furthermore, aBSREL analysis detected positively selected codons in three plethodontid branches but not in *Onychodactylus*. One of these three branches corresponded to the most recent common ancestor, and the others corresponded with the most recent common ancestors of direct-developing branches within Hemidactyliinae. The positive selection of mitochondrial protein-coding genes in Plethodontidae is probably associated with the evolution of direct development.

## 1. Introduction

Most eukaryotic organisms require oxygen to produce energy by aerobic respiration, which is performed in mitochondria. Mitochondria in animal cells have their own genome, which encodes 13 protein components that contribute to four of the five enzyme complexes of the oxidative phosphorylation pathway [1]: NADH dehydrogenase subunits 1–6 and 4 L (*nad1*–*6* and 4 L), cytochrome b (*cytb*), cytochrome c oxidase subunits 1–3 (*cox1*–*3*), and ATPase F0 subunit 6 and ATPase F0 subunit 8 (*atp6* and *atp8*). These mitochondrial protein subunits interact with nuclear-encoded proteins and are involved in aerobic respiration and ATP synthesis.

It had classically been believed that most of the variation in mitochondrial protein-coding genes is selectively neutral (meaning less convergent nucleotide substitution). Thus, these genes were frequently examined to infer phylogenetic relationships among populations or species [2]. It has also been generally accepted that mitochondrial DNA (mtDNA) is under strong functional constraints [3]. However, in animal taxa, the selective forces on mitochondrial protein-coding genes have been suggested to vary due to several factors, such as ambient oxygen environment [4], locomotive performance [5, 6], and life history patterns [7, 8]. In addition, accumulated evidence has shown that amino acid replacements in mitochondrial protein-coding genes are affected by adaptive evolution [9–11]. Such a process can be associated with metabolic performance [12, 13] and/or the animal's habitat/environment [14–17].

Lungs are the primary organ of the respiratory system in vertebrates and are almost ubiquitous among extant tetrapods. However, in several amphibians, the lungs have degenerated or been lost. Lungless taxa are present in all three amphibian orders: Anura, Gymnophiona, and Caudata [18–20]. In caudates (salamanders and newts), lunglessness has evolved in two distinct lineages: the family Plethodontidae and the genus *Onychodactylus* (belonging to the family Hynobiidae). Plethodontidae is the largest family of salamanders and consists of two subfamilies: Hemidactyliinae and Plethodontinae. Similar to most amphibians, some plethodontids have a biphasic life cycle, comprising an aquatic free-swimming larval stage followed by metamorphosis into a terrestrial adult (e.g., *Desmognathus fuscus* and *Eurycea bislineata*; referred to as metamorphosing plethodontids here).

The other lungless salamanders from genus *Onychodactylus* also have this biphasic life cycle. However, most plethodontids have adapted to terrestrial environments. They mate and lay eggs on land, and the juveniles hatch in the adult form without a free-swimming larval stage (direct development), enabling them to spend their entire lives only on land. Although plethodontids can also breathe through the buccopharyngeal cavity, they are highly dependent on cutaneous gas exchange for respiration [21]. In terms of functional aspects, Full et al. [22] showed that during exercise, maximum oxygen consumption and endurance were limited in plethodontids compared with those in lunged salamanders. However, the impact of lung loss on the mitochondrial genome remains unclear.

It was proposed that the lung loss in amphibians (such as the Bornean frog, *Barbourula kalimantanensis*, and Eiselt's caecilian, *Atretochoana eiselti*) would have prevented individuals from drifting downstream in mountain stream habitats, where the positive buoyancy due to the lungs would be maladaptive [19, 23]. This hypothesis was initially proposed for the family Plethodontidae [24–27]. This scenario is most likely associated with the reduced importance of lungs due to cool, fast-flowing, and oxygen-rich waters, assuming that plethodontids originated in the Appalachian Mountains. However, based on a geological perspective, Ruben and Boucot [28] insisted that such streams were not present in the Appalachian region at the time of the evolution of plethodontids. Another hypothesis proposed that the lunglessness of plethodontids evolved along with a behavioral shift, in which the courtship and mating behaviors of ancestral plethodontids stopped occurring in water and instead occurred on land [29]. This scenario assumes that the energetic cost of reproductive activities on land was lower than that in water. According to this assumption, salamanders require a lower amount of oxygen for reproductive activities, leading to reduced functional importance of lungs. Despite the distinct evolutionary hypotheses between these scenarios, oxygen requirements are considered to be among the important factors in the process of lung reduction; hence, mitochondrial performance should also be important.

mtDNA is a tractable system for investigating the local adaptation of organisms associated with different ecological traits and respiration environments. In salamanders, lung loss and behavioral change are probably related to mitochondrial performance in terms of energy production. In the present study, we investigated the evolution of mitochondrial protein-coding genes in salamanders to clarify whether selective constraints are involved in lunglessness. We further compared the selective constraints between direct-developing and metamorphosing plethodontids to understand the effect of terrestrial adaptation, as proposed by Reagan and Verrell [29]. Finally, we examined the occurrence of positive selection in mitochondrial genes in lungless salamanders to analyse the evolution of mitochondrial genomes associated with lunglessness in Caudata.

## 2. Materials and Methods

### 2.1. Molecular Data

We collected 86 mitochondrial genome sequences of caudates from the National Center for Biotechnology Information Reference Sequence (RefSeq) database (https://www.ncbi.nlm.nih.gov/refseq/) in April 2018. Because the sequences of several genera were not registered in RefSeq, we downloaded seven additional sequences from the GenBank database (https://www.ncbi.nlm.nih.gov/genbank/). In the preliminary selection analyses, calculation by the codeml program of the Phylogenetic Analysis by Maximum Likelihood (PAML) 4 package [30] took a very long time and sometimes did not complete due to the large number of sequences analysed. We removed one-third of the sequences (31/93) from genera containing more than two species to avoid this problem. The final dataset (62 sequences) covered all nine families of the order Caudata: Ambystomatidae, Amphiumidae, Cryptobranchidae, Hynobiidae, Plethodontidae, Proteidae, Rhyacotritonidae, Salamandridae, and Sirenidae. This dataset included 41 lunged salamanders, 2 *Onychodactylus* species, 13 direct-developing plethodontids, and 6 metamorphosing plethodontids.

We analysed four nuclear genes unrelated to mitochondrial function to test whether the similar selective patterns of the plethodontid mitochondrial genes are observed in the nuclear genes; we considered the following genes: brain-derived neurotrophic factor (*bdnf*), proopiomelanocortin (*pomc*), and recombination activating proteins 1 and 2 (*rag1* and *2*). The nuclear gene data did not cover all the 62 taxa, but each gene dataset covered at least five families of the order Caudata. The number of taxa included in each nuclear gene dataset was 28 (*bdnf*), 38 (*pomc*), 42 (*rag1*), and 29 (*rag2*) of the 62 analysed taxa. Of this nuclear dataset, the number of lunged salamander taxa was 15 (*bdnf*), 24 (*pomc*), 27 (*rag1*), and 20 (*rag2*). Each dataset included eight (*bdnf*), nine (*pomc*), nine (*rag1*), and four (*rag2*) taxa of direct-developing plethodontids and five (*bdnf*), five (*pomc*), six (*rag1*), and five (*rag2*) taxa of metamorphosing plethodontids. The taxa and accession numbers of the mitochondrial and nuclear sequences are provided in Supplementary Table S1.

### 2.2. Sequence Alignments and Phylogenetic Analyses

According to the inferred amino acid sequences translated by the portable version of TranslatorX version 1.1 [31] with the vertebrate mitochondrial genetic code, we aligned the mitochondrial protein-coding gene sequences using Multiple Alignment of the Fast Fourier Transform program [32]. We removed ambiguous alignment sites using Gblocks 0.91b [33] to exclude all gap-containing sites. We aligned nucleotide sequences of the four nuclear genes using the standard genetic code option. We used the corrected alignment of each mitochondrial and nuclear protein-coding gene for subsequent selection analyses.

We used the alignment data of 13 mitochondrial protein-coding genes for phylogenetic analysis. We checked the homogeneity of the nucleotide composition among taxa in each codon position of each gene using the chi-square test in Phylogears2 [34]. Because the nucleotide composition of the third codon positions of all genes, except *atp8*, significantly deviated from homogeneity (*P* < 0.001), we removed the third codon positions of all genes from the phylogenetic analysis. Finally, we used 6,888 alignment sites of the first and second codon positions of 13 genes (26 partitions) for phylogenetic analysis. We also adopted a partition model in the phylogenetic analyses. To determine the best partitioning scheme and substitution model for the suggested partitions, we used the greedy strategy [35] implemented in ModelFinder [36] in IQ-TREE version 1.6.3 [37]. The best partition scheme and substitution model for each partition are shown in Supplementary Table S2. We reconstructed the salamander phylogeny using the maximum likelihood (ML) and Bayesian inference (BI) methods. We performed ML analysis using IQ-TREE version 1.6.3 and evaluated the resultant ML tree's robustness using a nonparametric bootstrap method with 1,000 replicates. We inferred the BI tree from MrBayes version 3.2.6 [38]. We performed a run of 10 million generations, sampling every 1,000 generations and removing the initial 10% of samples as burn-in in the Markov chain Monte Carlo (MCMC) analysis. We confirmed the chains' convergence to stationarity by checking the effective sample size scores of all MCMC statistic parameters larger than 200 using Tracer version 1.6 [39]. We evaluated the robustness of the resultant BI tree using Bayesian posterior probabilities.

### 2.3. Tests of Selection

We performed three distinct selection approaches using the codeml program of the PAML 4 package [30]. These analyses by codeml were automated using LMAP version 1.0.2 [40].

First, we performed site model analysis to investigate the selective forces on each mitochondrial gene throughout the caudate phylogeny. In site model analysis, variation is estimated in *ω* value (=dN/dS ratio), i.e., the ratio of nonsynonymous (dN) to synonymous (dS) substitution rates, under the assumption that *ω* values vary among sites in the alignments throughout a phylogeny [41, 42]. We first compared one ratio model (M0: *ω* value are constant among sites) with a discrete model (M3: each site belongs to one of three different *ω* categories) for each gene to elucidate homogeneity in *ω* value among the sites. Next, we conducted tests to detect sites with *ω* > 1 using the following comparison: a nearly neutral model (M1a: there are two *ω* categories, one is restricted to 0 < *ω* < 1 and the other is *ω* = 1) vs. a positive selection model (M2a: there are three *ω* categories, two are restricted like M1a and the other is *ω* > 1) and a beta and *ω* = 1 model (M8a: there are two *ω* categories, one is drawn from beta distribution [0 < *ω* < 1] and the other is *ω* = 1) vs. a beta and *ω* > 1 model (M8: there are two *ω* categories, one is drawn from a beta distribution [0 < *ω* < 1] and the other is *ω* > 1). The names of these models were taken with reference to the PAML manual [30]. We applied the initial kappa value and branch length based on the results of preliminary analyses using the M0 model for each gene. The significance of the resultant ML values of these site models was tested using likelihood ratio tests (LRTs).

Second, we used the branch models [43, 44] to explore the difference in *ω* values associated with lunglessness. We calculated the *ω* values of plethodontids, *Onychodactylus*, and lunged salamanders for each gene. We tested the statistical significance of the difference in *ω* values between plethodontids and lunged salamanders or between *Onychodactylus* and lunged salamanders using LRT against their nested models. This procedure allows the *ω* values to vary between plethodontids and other salamanders or between *Onychodactylus* and others. Furthermore, we performed branch model analyses to investigate the difference of *ω* values between the biphasic and terrestrial life history of plethodontids by comparing *ω* values among lunged salamanders and direct-developing and metamorphosing plethodontids. Because the timing of transitions from biphasic life history to direct development has remained unclear [45, 46], we compared the *ω* values of tip branches only by assuming that all internal branches have a single *ω* value in the phylogeny. Statistical significance was tested in the same manner. We calculated the *ω* values of plethodontids, *Onychodactylus*, and lunged salamanders for each gene and compared the results with the above analyses, including internal branches, to check the reliability of the tip branch comparison.

The results of branch model analysis can be misleading for testing the relaxation of selective constraints because the *ω* value is affected by intensified positive selection. To avoid this problem, we used RELAX [47] implemented in HyPhy version 2.5.1 [48]. RELAX is used to test for relaxed or intensified selection using the parameter *k*, which modulates the degree to which *ω* categories deviate from selective neutrality (*ω* = 1). When selection is relaxed in test branches, the distribution of *ω* categories is close to 1 compared with that of reference branches, represented as *k* < 1. Inversely, the distribution of *ω* categories is far from 1, represented as *k* > 1, when selection is intensified in the test branches. We tested significance using an LRT, which compares the null model (*k* is fixed to 1) to the alternative model (*k* is a variable parameter). We performed five comparisons: (1) plethodontids vs. lunged salamanders, (2) *Onychodactylus* vs. lunged salamanders, (3) direct-developing plethodontids vs. lunged salamanders, (4) metamorphosing plethodontids vs. lunged salamanders, and (5) direct-developing plethodontids vs. metamorphosing plethodontids, corresponding to the branch model analyses.

We applied the same procedure of the above branch model and RELAX analyses for the four nuclear genes, excluding the execution of comparison (2), because we found no significant differences in the selective pattern of the mitochondrial genes, except for *cytb* between *Onychodactylus* vs. lunged salamanders. The tree topology used in the nuclear gene analyses was the same as that reconstructed from the mitochondrial gene data.

Finally, we examined the positive selection on mitochondrial protein-coding genes. To detect branches with positively selected codon sites, we used aBSREL [49] implemented in HyPhy version 2.5.1. For the aBSREL analysis, we used the default settings and resultant ML phylogeny for each gene. We assessed the statistical significance using LRT. The family-wise error rate due to multiple comparisons was controlled by the Holm–Bonferroni sequential rejection procedure implemented in aBSREL.

We further analysed the lungless branches identified by aBSREL using the branch-site model [50, 51]. We performed this analysis using the codeml program of the PAML 4 package [30]. The branch-site model allows *ω* values to vary among both sites and branches. We compared the alternative model, which allows a subset of sites to have *ω* > 1 in the branch of interest, with the null model, which applies a restriction to *ω* ≤ 1 to detect the presence of positively selected sites. We assessed the statistical significance using LRT. When the alternative model was adopted, the Bayes empirical Bayes (BEB) approach was used to calculate the posterior probability (PP) that a site is under positive selection [50]. We fixed the kappa value for each gene according to the results of the above preliminary analyses. We compared amino acid chemical properties between lunged and lungless species to understand the effect of positive selection on the identified sites and represented this comparison using WebLogo 3 [52]. We then classified the chemical properties of each amino acid in line with the default scheme of WebLogo 3.

## 3. Results

### 3.1. Phylogenetic Analysis

**Figure 1:**
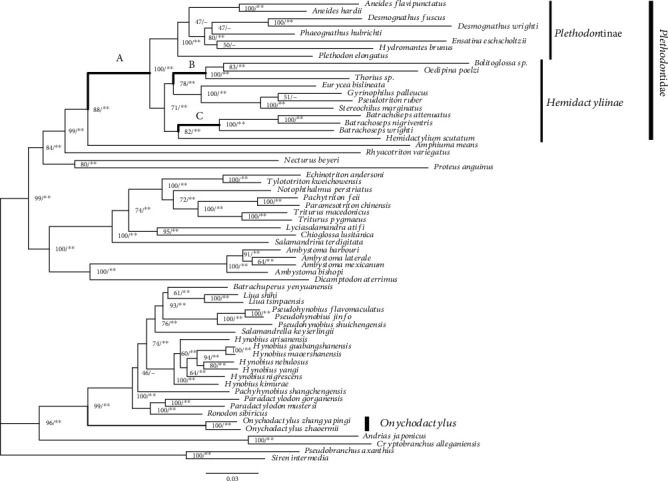
Caudate phylogeny inferred from 13 mitochondrial protein-coding genes (6,888 bp). The ML tree is shown. Bootstrap support and Bayesian posterior probability (∗∗>0.99, ∗>0.95) are denoted on each node. Species names in bold indicate direct-developing species. Branches with gray show lineages of lunged salamanders. Branches A, B, and C indicate the plethodontid branches with positively selected codon sites detected by aBSREL.

We reconstructed the phylogenetic relationships of salamanders using mitochondrial protein-coding gene data (Figure 1). The resultant tree topology based on the ML approach (log − likelihood = −98,518.40) was completely identical to that based on the BI approach (mean log − likelihood = −98,670.20). Statistical support was high in most nodes (ML bootstrap value > 60% and Bayesian PP > 95%). Although several nodes at lower taxonomic levels (e.g., phylogenetic relationships within the subfamilies Plethodontinae and Hemidactyliinae) were weakly supported, our phylogenetic results corroborated those of previous molecular phylogenetic studies of salamanders [53–55].

### 3.2. Selection Analysis

We used site model analyses to examine the general patterns of selective forces in each mitochondrial protein-coding gene throughout the caudate phylogeny (Supplementary Table S3). First, we compared the M0 model (constant *ω* model) with the M3 model (three-variable *ω* model). We rejected the M0 model (*P* < 0.01) in all genes. This result indicates that *ω* values varied among sites. Next, we compared M1a (two-variable *ω* model: *ω*_0_ < 1, *ω*_1_ = 1) with M2a (three-variable *ω* model: *ω*_0_ < 1, *ω*_1_ = 1, *ω*_2_ > 1) and M8a (beta distribution and *ω* = 1 model: *ω*_0_ follows a beta distribution and *ω*_1_ = 1) with M8 (beta distribution and *ω* > 1 model: *ω*_0_ follows a beta distribution and *ω*_1_>1) to detect positively selected sites throughout the phylogeny. For all genes, two null models (M1a and M8a), which assume no sites under positive selection, were not rejected (*P* > 0.1). These results indicate that negative selection was the dominant evolutionary trend in the mitochondrial genes of salamanders. We calculated the *ω* values of plethodontids, *Onychodactylus*, and lunged salamanders using a branch model (Table 1) to examine the selective constraints on lungless salamanders.

The results showed that the *ω* values of all mitochondrial protein-coding genes of plethodontids were higher than those of lunged salamanders. We detected statistical significance (*P* < 0.05) for nine genes (*atp6*, *cox1*–*3*, *nad1*, *2*, and *4–6*). In *Onychodactylus*, the *ω* values of five genes (*cox3*, *nad2*, *3*, *5*, and *6*) were higher than those of lunged salamanders, although the differences were not statistically significant. On the other hand, the *ω* value of *cytb* in *Onychodactylus* was significantly lower than that in lunged salamanders (*P* < 0.05). To confirm the robustness of branch model analyses without internal branches, we compared the *ω* values of terminal branches between plethodontids and lunged salamanders. The results showed that the *ω* values of 11 genes (*atp6*, *cox1*–*3*, *cytb*, and *nad1–6*) were higher in plethodontid salamanders than in lunged salamanders, and the differences were statistically significant in nine genes (*atp6*, *cox1*, *2*, *cytb*, *nad1*, *2*, and *4–6*) (Table S4). Although the *ω* values of four genes (*nad3–6*) in *Onychodactylus* were higher than those in the lunged taxa, the differences were not statistically significant. Thus, branch model analyses based on terminal branches resulted in similar trends as those based on both terminal and internal branches, that is, plethodontid salamanders have higher *ω* values than lunged salamanders.

Next, we applied the branch model analyses using terminal branches to compare *ω* values among direct-developing plethodontids, metamorphosing plethodontids, and lunged salamanders. For the terminal branch comparison between direct-developing plethodontids and lunged salamanders, the *ω* values of all genes, except *nad4L*, were higher in plethodontids, and the differences in *ω* values of nine genes (*atp6*, *cox1*–*3*, *nad1*, *2*, and *4*–*6*) were statistically significant (*P* < 0.01) (Table 2).

In the metamorphosing plethodontids, the *ω* values of 10 genes (*atp6*, *cox2*, *cytb*, *nad1*–*6*, and *4* L) were higher than those in lunged taxa, among which the *ω* values of five genes (*atp6*, *cytb*, *nad2*, *5*, and *6*) showed significance (*P* < 0.05). On comparing *ω* values between direct-developing plethodontids and metamorphosing plethodontids, eight genes (*atp8*, *cox1*–*3*, *nad1*, *2*, *4*, *5*) had higher *ω* values in direct-developing branches, among which four (*cox1*–*3*, *nad4*) genes showed significance (*P* < 0.05). There was no statistically significant difference in *ω* value between metamorphosing plethodontids and direct-developing taxa. These results indicate that direct-developing plethodontids exhibit potentially relaxed purifying selection compared with metamorphosing plethodontids.

The selective constraints, that is, the degree to which different *ω* categories diverged from neutrality (*ω* = 1), were further analysed using RELAX software. When comparing plethodontids and lunged salamanders, the selective constraints of all genes were significantly relaxed (*k* < 1, *P* < 0.05) in plethodontids, except that of *atp8* and *nad4L* (*k* < 1, *P* > 0.05) (Table 1). In the comparison between *Onychodactylus* and lunged salamanders, almost no genes were significant (*P* > 0.05), except *cytb* (*k* > 1, *P* < 0.05). When we compared selective constraints between direct-developing plethodontids and lunged salamanders, all genes exhibited significant relaxation of selective constraints (*k* < 1, *P* < 0.05), except *atp8* and *nad4L* (*k* ≥ 1, *P* > 0.05) in direct-developing plethodontids (Table 2). On the other hand, only three genes (*nad2*, *5*, *6*) exhibited significant relaxation in metamorphosing plethodontids compared with that in lunged species (*k* < 1, *P* < 0.05), but we did not detect significant differences in the other genes (*P* > 0.05). On comparing direct-developing and metamorphosing plethodontids, five genes (*cox1*–*3*, *nad4*, *5*) showed significant relaxation of selective pressure in direct-developing species (*k* < 1, *P* < 0.05), but we did not detect significant differences in other genes between these groups (*P* > 0.05). These results were almost consistent with the findings of branch model analyses and confirmed the trend that direct-developing plethodontids exhibited more relaxed selection in mitochondrial protein-coding genes than metamorphosing plethodontids.

For the nuclear datasets, branch model analyses with internal branches estimated that the *ω* values of three genes (*pomc*, *rag1*, and *rag2*) were higher in plethodontids than in lunged salamanders, although the difference was significant only in *rag1* (Table 3). RELAX analyses with internal branches showed significant relaxed selective constraints in *rag1* and *rag2*. Without the internal branches, neither branch model nor RELAX analyses resulted in a significant difference between plethodontids and lunged salamanders. In comparisons among direct-developing plethodontids, metamorphosing plethodontids, and lunged salamanders, there was no significant difference in selective constraints, except for the comparison between metamorphosing plethodontids and lunged salamanders by the branch model analysis of *pomc* (showing high *ω* in lunged salamanders). Consequently, the estimated selective pattern of the nuclear genes was not consistent with that of the mitochondrial genes. Specifically, the selective constraints of most mitochondrial genes were relaxed in direct-developing plethodontids compared with that in lunged salamanders, but there were no significant changes in the selective patterns of nuclear genes between these taxa.

aBSREL analysis detected eight distinct branches with positive selection from five genes (*P* < 0.05) as follows: most recent common ancestor (MRCA) of Plethodontidae (*nad4*, *5*); MRCA of *Batrachoseps* (*nad3*); MRCA of *Thorius*, *Oedipina*, and *Bolitoglossa* (*nad6*); MRCA of Sirenidae (*nad5*); MRCA of Rhyacotritonidae, Amphiumidae, and Plethodontidae (*cytb*); MRCA of Proteidae (*nad4*); MRCA of Salamandridae (*nad1*, *2*, *4*, *5*); and MRCA of *Triturus*, *Pachytriton*, and *Paramesotriton* (*atp6*). In this analysis, none of the *Onychodactylus* branches showed significant positive selection in any gene. For the plethodontid branches in which we detected significant positive selection in the above analysis, we further examined the positively selected codon sites by branch-site model analyses using the codeml program (Table 4). We conducted LRT between an alternative model (a subset of sites under positive selection) and a null model (no sites under positive selection) to test the presence of positively selected sites in each gene. The alternative model was significantly supported in *nad4* and *nad5* for the ancestral plethodontids, which coincided with the results of the aBSREL analysis. However, null models were not rejected in *nad3* for the MRCA of *Batrachoseps* and *nad6* for the MRCA of *Thorius*, *Oedipina*, and *Bolitoglossa*. In *nad4* and *5* for ancestral plethodontids, we calculated the posterior probabilities of positively selected sites using the BEB procedure implemented in the codeml program. In total, we estimated two codon sites (40 and 352) of *nad4* and three codon sites (23, 241, and 491) of *nad5* to be under positive selection in the ancestral plethodontid lineage (PP > 0.95).

**Figure 2:**
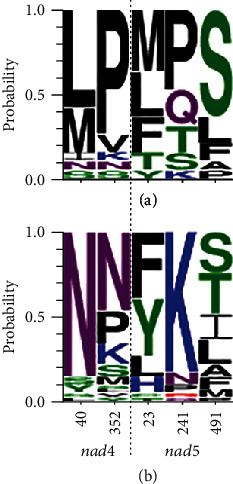
Amino acid composition of positively selected sites in Plethodontidae (a) and lunged salamanders (b). The color scheme of each amino acid represents the chemical properties, as determined using WebLogo 3: polar (green), neutral (purple), basic (blue), acidic (red), and hydrophobic (black).

On comparing amino acid chemical properties between Plethodontidae and lunged salamanders, we found that positively selected sites of *nad4* in lunged salamanders were highly occupied by Asn, which represents neutrality (Figure 2). However, the major amino acids of these sites in Plethodontidae were hydrophobic amino acids (i.e., Leu, Met, and Pro). In positively selected sites of *nad5*, most amino acids in Plethodontidae were hydrophobic (i.e., Leu, Met, Phe, and Pro), whereas in lunged salamanders, most were basic (i.e., Lys) at site 241 and polar (i.e., Tyr, Ser, and Thr) and hydrophobic (i.e., Phe, Leu, and Ile) at sites 23 and 491.

## 4. Discussion

### 4.1. Selective Constraints on Mitochondrial Protein-Coding Genes in Salamanders

We investigated natural selection trends in mitochondrial protein-coding genes in salamanders, with a particular focus on lungless taxa. Site model analyses (M1a vs. M2a and M8a vs. M8) showed *ω* < 1 for all genes throughout the salamander phylogeny, indicating that these genes have generally evolved under purifying selection. This result is consistent with the general evolutionary pattern of mitochondrial protein-coding genes in vertebrates (e.g., [56]).

Our branch model and RELAX analyses indicated that plethodontid salamanders tend to have higher *ω* values in mitochondrial protein-coding genes than other taxa, while *Onychodactylus* species showed a similar trend as that of lunged salamanders. Upon further analyses within plethodontids, we showed that direct-developing species have relaxed selective constraints in most genes (9 and 11 genes based on branch model and RELAX analyses, respectively) compared with that in lunged salamanders, although there was relaxed selective constraints in only three genes (*nad2*, *5*, and *6*) in metamorphosing plethodontids. These results suggest that lunglessness is less correlated with selective constraints on the mitochondrial genome in salamanders. Instead, we found that the relaxation of selective constraints in mitochondrial protein-coding genes of plethodontids is highly associated with direct-developing branches, for which there are two possible explanations. One possibility is due to energy requirements. Relaxed selection of mitochondrial protein-coding genes has been observed in specific groups that were hypothesized to have lower energy requirements in amphibians [57], birds [6], fish [8], and insects [5]. Reagan and Verrell [29] proposed that the energetic cost of reproductive activities on land is lower than that in water for salamanders. Furthermore, a previous study focusing on *Ambystoma maculatum* showed that the swimming performance of gravid females was lower than that of males and postgravid females [58], implying that the energetic cost of reproduction was higher in an aquatic than in a terrestrial environment. Direct-developing plethodontids that reproduce on land might be released from such energetic constraints, leading to relaxed selection on mitochondrial genes.

Another explanation for the relaxation of selective constraints in direct-developing plethodontids is the effect of genetic drift. In general, direct-developing species have a relatively small clutch size. This feature may lead to smaller population sizes, increasing the susceptibility to deleterious mutations due to genetic drift. This suggestion was supported by a study of mammalian mitochondrial genes [59], which showed that mammals with a small population size have accumulated more nonsynonymous nucleotide substitutions relative to the number of synonymous substitutions. If the latter hypothesis is true, genetic drift would affect not only mitochondrial but also nuclear genes. However, our branch model and RELAX analyses did not show relaxation of selective constraints in all four nuclear genes of direct-developing plethodontids compared with that of lunged salamanders and metamorphosing plethodontids. Thus, our result suggests that differences in population size are not the primary cause of the relaxed pattern in direct-developing plethodontids.

### 4.2. Positive Selection of Mitochondrial Protein-Coding Genes

aBSREL analysis detected the presence of positive selection on eight branches from among all caudate lineages. These branches are almost consistent with the ancestral lineages of major salamander taxa (e.g., MRCAs of Proteidae, Salamandridae, and Sirenidae). Within the family Plethodontidae, MRCA of Plethodontidae had a significant positive selection in *nad4* and *nad5*, MRCA of *Batrachoseps* in *nad3*, and MRCA of three plethodontid genera (*Bolitoglossa*, *Oedipina*, and *Thorius*) in *nad6* (branches A, B, and C, respectively, Figure 1). Of these, the latter two branches were consistent with MRCAs of direct-developing plethodontids within Hemidactyliinae (Figure 1). Direct development is a unique trait in plethodontids in Caudata. Previous studies based on the reconstruction of ancestral traits showed that the developmental mode of MRCA of Plethodontidae is likely to direct development [60–62]. Positive selection in plethodontids appears to be congruent with the evolution of direct development.

Of the three plethodontid branches undergoing positive selection at some mitochondrial protein-coding genes, MRCA of Plethodontidae in *nad4* and *nad5* was validated in the branch-site model analyses. The branch-site model analyses identified five positively selected sites from *nad4* and *nad5* on the ancestral lineages of Plethodontidae by the BEB procedure. When we compared each site's amino acid composition, plethodontids were found to have more hydrophobic residues relative to that in lunged salamanders (Figure 2). An increase in the number of amino acid residues with high hydrophobicity contributes to protein stability via hydrophobic interaction [63]. Thus, the substitutions to hydrophobic amino acids observed in positively selected sites might suggest that positive selection contributed to higher protein stability.

### 4.3. Evolutionary Implications for Lungless Salamanders

The present study showed that the evolutionary pattern of mitochondrial protein-coding genes in Plethodontidae differed from that in another lungless taxon (*Onychodactylus*) and many other lunged salamanders. We found signals of positive selection and relaxed selection within Plethodontidae. These selective patterns in Plethodontidae were not associated with lung loss but with the evolution of direct development. In contrast, other lungless salamanders, such as those from *Onychodactylus*, have a biphasic life history and require cool underground flowing water for breeding [64, 65]. Such a feature is consistent with the major hypothesis for lunglessness evolution. Because there was no significant difference in selective constraints between *Onychodactylus* and lunged salamanders, *Onychodactylus* species would have access to sufficient oxygen due to their environment and/or gas exchange through their skin and buccopharyngeal cavity.

Recent studies using ancestral state reconstructions supported that the life history mode of ancestral Plethodontidae is direct development [60–62]. These results contradict the major hypothesis that lunglessness in salamanders evolved as an adaptation to a fast-flowing stream environment [25–27] because this adaptation is focused on aquatic free-swimming larvae [24]. Because direct-developing plethodontids do not need an aquatic environment, another hypothesis proposed by Reagan and Verrell [29] that lunglessness originated from terrestrial adaptation is more reasonable. Reagan and Verrell's hypothesis supposed that reproductive activities (i.e., migration to breeding site, courting, mating, and laying eggs in water) are energetically costly for biphasic salamanders, such as the majority of Ambystomatidae and Salamandridae. Such terrestrial adaptation would decrease salamanders' energy requirements, leading to low oxygen requirements followed by reduced importance of lungs. The results of the branch model and RELAX analyses suggest that genetic drift does not cause the relaxed selective constraints of mitochondrial genes observed in direct-developing plethodontids due to the reduced population size. If this relaxed selective constraint originates from relaxed functional constraints of mitochondrial proteins, the assumption of Reagan and Verrell's hypothesis would be supported. Further studies regarding mitochondrial protein-coding genes may shed light on the evolutionary history of plethodontid salamanders.

## Figures and Tables

**Table 1:** Results of branch model and RELAX analyses of mitochondrial genes with internal branches.

Comparison	Gene	Branch model				RELAX		
		-2 *Δ*lnL (d.f. = 1)	*P*	*ω*1	*ω*2	-2 *Δ*lnL (d.f. = 1)	*P*	*k*
Plethodontidae vs. lunged salamanders	*atp6*	-11.0074	*0.001*	0.046	0.033	-22.0306	*0.000*	0.722
	*atp8*	-0.7743	0.379	0.142	0.138	-0.2942	0.588	0.684
	*cox1*	-45.3786	*0.000*	0.013	0.007	-30.5404	*0.000*	0.842
	*cox2*	-42.3632	*0.000*	0.035	0.017	-50.8092	*0.000*	0.625
	*cox3*	-3.9068	*0.048*	0.033	0.028	-6.8323	*0.009*	0.891
	*cytb*	-1.4472	0.229	0.036	0.033	-4.4370	*0.035*	0.849
	*nad1*	-4.6464	*0.031*	0.030	0.025	-6.3163	*0.012*	0.852
	*nad2*	-36.1991	*0.000*	0.069	0.045	-28.5936	*0.000*	0.706
	*nad3*	-1.6005	0.206	0.054	0.046	-7.5547	*0.006*	0.784
	*nad4*	-16.1672	*0.000*	0.049	0.038	-11.2847	*0.001*	0.886
	*nad4L*	-0.4267	0.514	0.043	0.039	-0.2034	0.652	0.953
	*nad5*	-19.6304	*0.000*	0.056	0.044	-44.8124	*0.000*	0.759
	*nad6*	-21.7759	*0.000*	0.072	0.040	-29.7902	*0.000*	0.614

*Onychodactylus* vs. lunged salamanders	*atp6*	-0.9611	0.327	0.024	0.033	-0.0133	0.908	1.036
	*atp8*	-0.0915	0.762	0.105	0.138	-0.3936	0.530	7.676
	*cox1*	-1.2064	0.272	0.005	0.007	-1.2101	0.271	1.111
	*cox2*	-0.0705	0.791	0.016	0.017	-0.1654	0.684	1.019
	*cox3*	-0.1605	0.689	0.031	0.028	-0.9278	0.335	0.876
	*cytb*	-4.7672	*0.029*	0.020	0.033	-5.2470	*0.022*	1.477
	*nad1*	-0.9503	0.330	0.019	0.025	-0.5157	0.473	1.103
	*nad2*	-0.4529	0.501	0.053	0.045	-0.1290	0.719	0.968
	*nad3*	-0.0766	0.782	0.051	0.046	-1.4217	0.233	1.115
	*nad4*	-0.0001	0.994	0.038	0.038	-0.6303	0.427	1.129
	*nad4L*	-0.7078	0.400	0.025	0.039	-0.8172	0.366	1.337
	*nad5*	-0.1328	0.716	0.047	0.044	-0.0149	0.903	1.017
	*nad6*	-0.5708	0.450	0.054	0.040	-1.4766	0.224	0.690

Values with italic showed *P* < 0.05. *ω*1 and *ω*2 indicate *ω* values in test and reference branches, respectively. Both *ω* values were calculated based on alternative models.

**Table 2:** Results of branch model and RELAX analyses of mitochondrial genes without internal branches.

Comparison	Gene	Branch model				RELAX		
		-2 *Δ*lnL (d.f. = 1)	*P*	*ω*1	*ω*2	-2 *Δ*lnL (d.f. = 1)	*P*	*k*
Plethodontidae DD vs. lunged salamanders	*atp6*	-7.4708	*0.006*	0.048	0.033	-14.0500	*0.000*	0.773
	*atp8*	-0.0262	0.871	0.147	0.139	-0.0706	0.790	1.222
	*cox1*	-45.1340	*0.000*	0.015	0.007	-34.4024	*0.000*	0.826
	*cox2*	-45.5633	*0.000*	0.044	0.018	-51.4044	*0.000*	0.555
	*cox3*	-6.7130	*0.010*	0.039	0.028	-8.6419	*0.003*	0.842
	*cytb*	-2.2026	0.138	0.035	0.030	-4.8904	*0.027*	0.827
	*nad1*	-8.5458	*0.003*	0.032	0.023	-10.9626	*0.001*	0.762
	*nad2*	-33.2628	*0.000*	0.072	0.041	-32.1149	*0.000*	0.652
	*nad3*	-0.2201	0.639	0.053	0.049	-9.6473	*0.002*	0.689
	*nad4*	-18.0457	*0.000*	0.055	0.038	-17.7786	*0.000*	0.826
	*nad4L*	-0.0002	0.989	0.040	0.040	0.0000	1.000	1.000
	*nad5*	-16.8708	*0.000*	0.055	0.040	-38.3770	*0.000*	0.803
	*nad6*	-11.6646	*0.001*	0.073	0.042	-13.0165	*0.000*	0.705

Plethodontidae MM vs. lunged salamanders	*atp6*	-4.4268	*0.035*	0.049	0.033	-1.6165	0.204	0.859
	*atp8*	-0.4924	0.483	0.101	0.139	-1.4104	0.235	1.759
	*cox1*	-0.0082	0.928	0.007	0.007	-0.5711	0.450	1.047
	*cox2*	-2.5963	0.107	0.024	0.018	-2.2037	0.138	0.914
	*cox3*	-1.9094	0.167	0.022	0.028	-1.4144	0.234	1.132
	*cytb*	-4.5094	*0.034*	0.040	0.030	-1.4137	0.234	0.800
	*nad1*	-1.2678	0.260	0.027	0.023	-0.3337	0.564	0.967
	*nad2*	-5.3517	*0.021*	0.056	0.041	-5.3525	*0.021*	0.851
	*nad3*	-2.4007	0.121	0.071	0.049	-1.9701	0.160	0.763
	*nad4*	-0.0627	0.802	0.039	0.038	-0.6735	0.412	1.057
	*nad4L*	-0.0001	0.994	0.040	0.040	-0.1075	0.743	1.065
	*nad5*	-4.5264	*0.033*	0.050	0.040	-13.2843	*0.000*	0.834
	*nad6*	-6.8491	*0.009*	0.077	0.042	-9.0366	*0.003*	0.679

Plethodontidae DD vs. Plethodontidae MM	*atp6*	-0.0041	0.949	0.048	0.049	-0.4736	0.491	0.832
	*atp8*	-0.5788	0.447	0.147	0.101	-1.4551	0.228	0.617
	*cox1*	-17.9793	*0.000*	0.015	0.007	-18.0031	*0.000*	0.573
	*cox2*	-8.2538	*0.004*	0.044	0.024	-7.8247	*0.005*	0.647
	*cox3*	-8.4264	*0.004*	0.039	0.022	-10.0902	*0.001*	0.614
	*cytb*	-0.8062	0.369	0.035	0.040	-0.7953	0.373	0.892
	*nad1*	-0.8558	0.355	0.032	0.027	-1.6064	0.205	0.824
	*nad2*	-3.1405	0.076	0.072	0.056	-3.0969	0.078	0.835
	*nad3*	-1.2157	0.270	0.053	0.071	-0.3625	0.547	0.905
	*nad4*	-6.4172	*0.011*	0.055	0.039	-12.1234	*0.000*	0.809
	*nad4L*	-0.0003	0.987	0.040	0.040	-0.4868	0.485	0.846
	*nad5*	-0.6011	0.438	0.055	0.050	-49.3683	*0.000*	0.944
	*nad6*	-0.0398	0.842	0.073	0.077	-0.6219	0.430	1.084

Values with italic showed *P* < 0.05. *ω*1 and *ω*2 indicate *ω* values in test and reference branches, respectively. Both *ω* values were calculated based on alternative models. DD and MM represent direct-developing and metamorphosing taxa, respectively.

**Table 3:** Results of branch model and RELAX analyses of nuclear genes.

Comparison	Gene	Branch model				RELAX		
		-2 *Δ*lnL (d.f. = 1)	*P*	*ω*1	*ω*2	-2 *Δ*lnL (d.f. = 1)	*P*	*k*
(All branches)								

Plethodontidae vs. lunged salamanders	*bdnf*	0.0317	0.859	0.020	0.021	0.0742	0.785	1.033
	*pomc*	1.5407	0.215	0.087	0.084	0.0440	0.834	1.035
	*rag1*	9.8984	*0.002*	0.073	0.046	9.2968	*0.002*	0.670
	*rag2*	1.5442	0.214	0.173	0.142	7.0458	*0.008*	0.708

(Terminal branches)								

Plethodontidae vs. lunged salamanders	*bdnf*	0.2244	0.636	0.017	0.024	0.4279	0.513	1.111
	*pomc*	0.8963	0.344	0.078	0.103	1.2456	0.264	1.424
	*rag1*	2.7077	0.100	0.079	0.058	2.7967	0.094	0.790
	*rag2*	1.8219	0.177	0.217	0.164	2.4296	0.119	0.796

Plethodontidae DD vs. lunged salamanders	*bdnf*	0.5339	0.465	0.012	0.022	0.7742	0.379	1.183
	*pomc*	0.0061	0.938	0.105	0.103	0.3638	0.546	1.313
	*rag1*	1.6841	0.194	0.077	0.058	1.6312	0.202	0.443
	*rag2*	0.3989	0.528	0.193	0.164	0.6024	0.438	0.811

Plethodontidae MM vs. lunged salamanders	*bdnf*	-0.0371	—	0.026	0.022	0.0037	0.951	1.013
	*pomc*	5.7446	*0.017*	0.041	0.103	1.8583	0.173	1.607
	*rag1*	1.8707	0.171	0.082	0.058	2.6868	0.101	0.807
	*rag2*	2.3532	0.125	0.249	0.164	3.1834	0.074	0.672

Plethodontidae DD vs. Plethodontidae MM	*bdnf*	0.4522	0.501	0.012	0.026	0.3311	0.565	1.229
	*pomc*	3.4992	0.061	0.105	0.041	0.4250	0.514	0.816
	*rag1*	0.0406	0.840	0.077	0.082	0.5278	0.468	1.106
	*rag2*	0.6134	0.434	0.193	0.249	0.6722	0.412	1.483

Values with italic showed *P* < 0.05. *ω*1 and *ω*2 indicate *ω* values in test and reference branches, respectively. Both *ω* values were calculated based on alternative models. DD and MM represent direct-developing and metamorphosing branches, respectively. All and terminal branches with parentheses denote the comparison of all (internal and terminal) branches and only terminal branches, respectively. Dash showed that *P* values could not be calculated due to the lower lnL of the alternative model than the null model.

**Table 4:** Results of branch-site model analyses in Plethodontidae.

Branch	Gene	-2 *Δ*lnL (d.f. = 1)	*P*	Positively selected sites
A	*nad4*	-8.3999	*0.004*	40∗
				352∗
	*nad5*	-9.5527	*0.002*	23∗∗
				241∗∗
				491∗

B	*nad6*	-0.0110	0.916	

C	*nad3*	0.0000	1.000	

Values with italic indicate *P* < 0.05. Positively selected sites were estimated by BEB analyses for genes which positive selection was detected by LRT. Only PP > 0.95 are shown. ∗ and ∗∗ represent PP > 0.95 and >0.99, respectively. Branch labels were referred to as Figure 1.

## Data Availability

Sequence data that support the findings of this study were deposited in the National Center for Biotechnology Information database (http://www.ncbi.nlm.nih.gov/). The list of accession numbers generated in this study is given in Supplementary Table S1.

## Supplementary Materials

Supplementary Materials We have provided additional table files as follows: Supplementary Table S1: mitochondrial genome sequences used in this study. Supplementary Table S2: best-fit partition scheme and substitution models for phylogenetic analyses. Supplementary Table S3: results of site model analysis. Supplementary Table S4: results of branch model analysis without internal branches.
