# Supplementary material for: Patterns of Natural Selection on Mitochondrial Protein-Coding Genes in Lungless Salamanders: Relaxed Purifying Selection and Presence of Positively Selected Codon Sites in the Family Plethodontidae
Source: Int J Genomics. 2021 Apr 9;2021:6671300. doi: 10.1155/2021/6671300 (PMC8053045; doi:10.1155/2021/6671300)
Supplement: Supplementary Materials — We have provided additional table files as follows: Supplementary Table S1: mitochondrial genome sequences used in this study. Supplementary Table S2: best-fit partition scheme and substitution models for phylogenetic analyses. Supplementary Table S3: results of site model analysis. Supplementary Table S4: results of branch model analysis without internal branches. [file 6671300.f1.pdf]

Supplementary Table S1 List of salamander taxa used in this study

Family	Species	Accession No.					Lungs	Life cycle in Plethodontidae
		Mt genome	BDNF	POMC	Rag1	Rag2		
Ambystomatidae	*Ambystoma barbouri*	NC_014568	KJ610094	KJ610458			+	
	Ambystoma bishopi	NC_027501					+	
	Ambystoma laterale	NC_006330					+	
	Ambystoma mexicanum	NC_005797			AY323752	KC165605	+	
	Dicamptodon aterrimus	GQ368657			KC165591	KC165611	+	
Amphiumidae	*Amphiuma means*	GQ368656		AY141896	AY650127	KC165604	+	
Cryptobranchidae	*Andrias japonicus*	NC_007446			AY583346		+	
	Cryptobranchus alleganiensis	GQ368662			AY650141		+	
Hynobiidae	*Batrachuperus yenyuanensis*	NC_012430	HM037740	HM037765	HM037715	KC165607	+	
	Hynobius arisanensis	NC_009335					+	
	Hynobius guabangshanensis	NC_013762	HM037743	HM037768	HM037718	KJ715336	+	
	Hynobius kimurae	NC_026032			KJ715369	KJ715332	+	
	Hynobius maoershanensis	NC_023789	HM037745	HM037770	HM037720		+	
	Hynobius nebulosus	NC_020650	HM037746	HM037771	AY650144		+	
	Hynobius nigrescens	NC_026033			KJ715356	KJ715337	+	
	Hynobius yangi	NC_013825					+	
	Liua shihi	NC_008078	HM037751	HM037776	HM037726	KJ715344	+	
	Liua tsinpaensis	NC_008081	HM037752	HM037777	HM037727	KJ715345	+	
	Onychodactylus zhangyapingi	NC_026853					-	
	Onychodactylus zhaoermii	NC_026854					-	
	Pachyhynobius shangchengensis	NC_008080	HM037754	HM037779	HM037729	KJ715349	+	
	Paradactylodon gorganensis	NC_008091	HM037755	HM037780	HM037730		+	
	Paradactylodon mustersi	NC_008090	HM037756	HM037781	HM037731	KJ715350	+	
	Pseudohynobius flavomaculatus	NC_020635	HM037758	HM037783	HM037733	KJ715346	+	
	Pseudohynobius jinfo	NC_026698					+	
	Pseudohynobius shuichengensis	NC_021001		HM037784	HM037734	KJ715347	+	
	Ranodon sibiricus	NC_004021	HM037760	HM037785	HM037735	KC165618	+	
	Salamandrella keyserlingii	NC_008082	KJ855091	KJ855092	AY650145	MF567484	+	
Plethodontidae	*Aneides flavipunctatus*	NC_006327	MF946481	MF946506			-	DD
	Aneides hardii	NC_006338	MF946486		EU275780	KC165603	-	DD
	Batrachoseps attenuatus	NC_006340	JF449372	KP900129	KM202870	KF911907	-	DD
	Batrachoseps nigriventris	NC_028184		KM202742	KM202918		-	DD
	Batrachoseps wrighti	NC_006333	JF449369	KM202864	KM203042		-	DD
	Bolitoglossa sp.	NC_006346					-	DD
	Desmognathus fuscus	NC_006339	EU275858	EU275812	EU275781	KF911911	-	MM
	Desmognathus wrighti	NC_006337		KR732363	AY691699	KF911913	-	DD
	Ensatina eschscholtzii	NC_006328	EU275862	EU275816	AY650119	KF911914	-	DD
	Eurycea bislineata	NC_006329	EU275861	JQ920726	AY691706	KC165612	-	MM
	Gyrinophilus palleucus	NC_028297			KY073116		-	MM
	Hemidactylium scutatum	NC_006342	EU275898	EU275852	AY691711	KF911917	-	MM
	Hydromantes brunus	NC_006345	EU275871	EU275825	HM797619		-	DD
	Oedipina poelzi	NC_006326					-	DD
	Phaeognathus hubrichti	NC_006344	EU275860	EU275814	AY691700		-	DD
	Plethodon elongatus	NC_006335	EU275882	EU275836	AY650120		-	DD
	Pseudotriton ruber	NC_006332	EU275900	EU275854	AY650123	KF911925	-	MM
	Stereochilus marginatus	NC_006325	EU275859	JQ920723	AY691713	KF911926	-	MM
	Thorius sp.	NC_006336					-	DD
Proteidae	*Necturus beyeri*	NC_023341		JX144998	JX145010	KC165613	+	
	Proteus anguinus	NC_023342		KC295576	AY650138	KC165615	+	
Rhyacotritonidae	*Rhyacotriton variegatus*	NC_006331	EU275869	EU275823	AY691693	KC165619	+	
Salamandridae	*Chioglossa lusitanica*	EU880308			AY583347		+	
	Echinotriton andersoni	NC_017870		HM462065			+	
	Lyciasalamandra atifi	NC_002756					+	
	Notophthalmus perstriatus	NC_028278					+	
	Pachytriton feii	NC_029345		KU375037	JX907980		+	
	Paramesotriton chinensis	NC_035008					+	
	Salamandrina terdigitata	EU880332		HQ915067			+	
	Triturus macedonicus	NC_015794					+	
	Triturus pygmaeus	NC_015796					+	
	Tylototriton kweichowensis	NC_029231	KY800640	KC733384			+	
Sirenidae	*Pseudobranchus axanthus*	GQ368660		JX145003	JX145015	KC165616	+	
	Siren intermedia	GQ368661		JX145005	AY583353	KC165620	+	

Supplementary Table S2 Best-fit partition scheme and substitution models

Partition	Gene + Codon position	Substitution model	
		IQTREE	MrBayes
1	atp6_1, atp8_1, nd1_1, nd3_1, nd4_1, nd4l_1	GTR+F+R5	GTR+F+I+G4
2	atp6_2, atp8_2, nd1_2, nd2_2, nd3_2, nd4_2, nd4l_2, nd5_2	TVM+F+R5	GTR+F+I+G4
3	co1_1, co3_2, cyb_1, cyb_2	TIM2+F+R5	GTR+F+I+G4
4	co1_2, co2_1	SYM+R4	SYM+I+G4
5	co2_2, co3_1	TPM2u+F+R2	F81+F+I+G4
6	nd2_1, nd5_1	GTR+F+R4	GTR+F+I+G4
7	nd6_1	TIM2+F+I+G4	GTR+F+I+G4
8	nd6_2	HKY+F+R3	HKY+F+I+G4

Numbers following G or R represent the number of categories.

Supplementary Table 3 Results of site model analysis

Gene		Null model (n.p.)	Alt. model (n.p.)	LRT	ω (M0)
ATP6		M0 (121)	M3 (126)		0.03724
	lnL	-18000.496357	-15630.310836	**0.000**	
		M1a (123)	M2a (125)		
	lnL	-16178.537504	-16178.53751	-	
		M7 (123)	M8 (125)		
	lnL	-15601.93418	-15601.93602	-	
		M8a (124)	M8 (125)		
	lnL	-15601.309362	-15601.93602	-	
ATP8		M0 (121)	M3 (126)		0.13866
	lnL	-1767.285964	-1612.522287	**0.000**	
		M1a (123)	M2a (125)		
	lnL	-1639.457606	-1638.80797	0.522	
		M7 (123)	M8 (125)		
	lnL	-1608.611551	-1606.365845	0.106	
		M8a (124)	M8 (125)		
	lnL	-1606.63545	-1606.365845	0.463	
COX1		M0 (121)	M3 (126)		0.00855
	lnL	-39900.319837	-31436.760677	**0.000**	
		M1a (123)	M2a (125)		
	lnL	-31973.925214	-31973.925214	1.000	
		M7 (123)	M8 (125)		
	lnL	-31424.381615	-31424.386685	-	
		M8a (124)	M8 (125)		
	lnL	-31421.370034	-31424.386685	-	
COX2		M0 (121)	M3 (126)		0.02328
	lnL	-16827.851769	-13929.963922	**0.000**	
		M1a (123)	M2a (125)		
	lnL	-14215.074489	-14215.074489	1.000	
		M7 (123)	M8 (125)		
	lnL	-13924.766613	-13924.768873	-	
		M8a (124)	M8 (125)		
	lnL	-13923.483977	-13924.768873	-	
COX3		M0 (121)	M3 (126)		0.02976
	lnL	-18429.076548	-15419.218485	**0.000**	
		M1a (123)	M2a (125)		
	lnL	-15948.945676	-15948.945676	1.000	
		M7 (123)	M8 (125)		
	lnL	-15394.600829	-15394.602917	-	
		M8a (124)	M8 (125)		
	lnL	-15393.444469	-15394.602917	-	
CYTB		M0 (121)	M3 (126)		0.03365
	lnL	-34165.175775	-29006.708198	**0.000**	
		M1a (123)	M2a (125)		
	lnL	-30125.289556	-30125.289556	1.000	
		M7 (123)	M8 (125)		
	lnL	-28981.353549	-28981.357319	-	
		M8a (124)	M8 (125)		

	lnL	-28980.765282	-28981.357319	-	
ND1		M0 (121)	M3 (126)		0.02645
	lnL	-26480.902137	-22321.408577	**0.000**	
		M1a (123)	M2a (125)		
	lnL	-23103.125516	-23103.125551	-	
		M7 (123)	M8 (125)		
	lnL	-22308.465961	-22308.468951	-	
		M8a (124)	M8 (125)		
	lnL	-22307.594155	-22308.468951	-	
ND2		M0 (121)	M3 (126)		0.05317
	lnL	-29851.10539	-26922.567855	**0.000**	
		M1a (123)	M2a (125)		
	lnL	-27692.028658	-27692.028658	1.000	
		M7 (123)	M8 (125)		
	lnL	-26895.343391	-26895.346431	-	
		M8a (124)	M8 (125)		
	lnL	-26894.934545	-26895.346431	-	
ND3		M0 (121)	M3 (126)		0.04871
	lnL	-10034.050928	-8580.749766	**0.000**	
		M1a (123)	M2a (125)		
	lnL	-8951.551042	-8951.551042	1.000	
		M7 (123)	M8 (125)		
	lnL	-8578.494095	-8578.495125	-	
		M8a (124)	M8 (125)		
	lnL	-8578.043563	-8578.495125	-	
ND4		M0 (121)	M3 (126)		0.0416
	lnL	-39574.003357	-34673.370898	**0.000**	
		M1a (123)	M2a (125)		
	lnL	-35843.649173	-35843.649173	1.000	
		M7 (123)	M8 (125)		
	lnL	-34622.498124	-34622.502231	-	
		M8a (124)	M8 (125)		
	lnL	-34621.459507	-34622.502231	-	
ND4l		M0 (121)	M3 (126)		0.03979
	lnL	-7907.917641	-6900.888554	**0.000**	
		M1a (123)	M2a (125)		
	lnL	-7149.575349	-7149.575349	1.000	
		M7 (123)	M8 (125)		
	lnL	-6897.612831	-6897.613671	-	
		M8a (124)	M8 (125)		
	lnL	-6897.535354	-6897.613671	-	
ND5		M0 (121)	M3 (126)		0.04813
	lnL	-51719.607871	-45408.281543	**0.000**	
		M1a (123)	M2a (125)		
	lnL	-47218.308293	-47218.308293	1.000	
		M7 (123)	M8 (125)		
	lnL	-45343.881613	-45343.886424	-	
		M8a (124)	M8 (125)		
	lnL	-45342.385343	-45343.886424	-	

ND6		M0 (121)	M3 (126)		0.05089
	lnL	-12177.525846	-10809.786492	**0.000**	
		M1a (123)	M2a (125)		
	lnL	-11159.398501	-11159.398501	1.000	
		M7 (123)	M8 (125)		
	lnL	-10784.246169	-10784.247429	-	
		M8a (124)	M8 (125)		
	lnL	-10783.79465	-10784.247429	-	

n.p. is the estimated number of parameters. Dash represents lnL of alternative model is lower than that of null model, and unable to perform LRT.

Table S4

Supplementary Table 4 Results of branch model analyses without internal branches.

Comparison	Gene	Branch model			
		-2 ΔlnL (d.f. = 1)	p-value	ω1	ω2
Plethodontidae	*atp6*	-10.0672	**0.002**	0.048	0.033
vs. lunged salamanders	*atp8*	-0.0467	0.829	0.132	0.139
	cox1	-28.4104	**0.000**	0.012	0.007
	cox2	-36.7897	**0.000**	0.038	0.018
	cox3	-2.1061	0.147	0.034	0.028
	cytb	-4.6570	**0.031**	0.036	0.030
	nad1	-7.6948	**0.006**	0.030	0.023
	nad2	-31.2956	**0.000**	0.067	0.041
	nad3	-1.3742	0.241	0.058	0.049
	nad4	-12.8570	**0.000**	0.050	0.038
	nad4L	-0.0004	0.983	0.040	0.040
	nad5	-19.2041	**0.000**	0.053	0.040
	nad6	-15.5252	**0.000**	0.074	0.042
Onychodactylus	*atp6*	-0.0221	0.882	0.035	0.038
vs. lunged salamanders	*atp8*	-0.1093	0.741	0.105	0.137
	cox1	-1.2962	0.255	0.003	0.009
	cox2	-0.0349	0.852	0.016	0.024
	cox3	-0.0006	0.980	0.029	0.030
	cytb	-0.1953	0.659	0.027	0.032
	nad1	-0.0785	0.779	0.021	0.026
	nad2	-0.1300	0.718	0.045	0.049
	nad3	-0.5475	0.459	0.069	0.051
	nad4	-1.4853	0.223	0.052	0.042
	nad4L	-0.0208	0.885	0.036	0.040
	nad5	-1.8241	0.177	0.053	0.044
	nad6	-0.6594	0.417	0.059	0.053

Values with bold showed $P < 0.05$. ω1 and ω2 indicate ω values in test and reference lineages, respectively.
